# Impact of credit access on farm performance: Does source of credit matter?

**DOI:** 10.1016/j.heliyon.2023.e19720

**Published:** 2023-09-09

**Authors:** Tri Haryanto, Wahyu Wisnu Wardana, Iqram Ramadhan Jamil, Annisaa Rizky Dwi Brintanti, Kabiru Hannafi Ibrahim

**Affiliations:** a Department of Economics, Faculty of Economics and Business, Universitas Airlangga, Indonesia; b Research Institute of Socio-Economic Development (RISED) Surabaya, Indonesia; c Department of Economics, Faculty of Economics and Business, Universitas Padjadjaran, Indonesia; d Faculty of Social and Management Sciences, Federal University Birnin Kebbi, Nigeria

**Keywords:** Agricultural credit, Formal and informal sources, Technical efficiency, Maize productivity, Agricultural development, Indonesia

## Abstract

Access to credit is crucial to improve farm performance as it allows farmers to procure inputs and technology. However, on the empirical front, evidence of the impact of agricultural credit access remains scanty. This study examines how access to credit from formal and informal sources influences the productivity and technical efficiency of maize farming in ten major maize-producing provinces in Indonesia. Secondary Food Crops Survey data by Statistics Indonesia were employed and analyzed using a quasi-experimental approach, i.e., the propensity score matching (PSM). The estimation shows that agricultural credit access improves farm performance. In fact, the ability to obtain credit from institutions increased productivity and technical efficiency more effectively than from informal sources. This study suggests that agricultural credit access remains relevant in Indonesia and needs to be improved continuously.

## 1 Introduction

The agricultural sector is often considered peripheral to the economic arena [1] when, in fact, it is central to the economy, especially during a crisis [2]. For instance, while economic sectors dropped amidst the COVID-19 pandemic in some countries, the agriculture sector grew about 2.2% in Indonesia and 3.5% in India [3,4]. Moreover, as the world population increases, food insufficiency and hunger risks also increase. The agriculture sector is crucial in maintaining food availability [5], so productivity and performance need to be increased.

Agriculture is a strategic sector in developing countries [6]; for example, in Indonesia, the agricultural sector contributed 9.85% of the total GDP in 2021 and absorbed 29.96% of the working population [7]. However, the performance of the agriculture sector in Indonesia is not optimal. For example, in the maize subsector, Indonesia's maize production is one of the ten largest in the world, but compared to other major producers, the productivity is relatively low. The United States Department of Agriculture [8] notes that the average of Indonesia's maize productivity from 2008 to 2018 was 2.81 tons per hectare, lower than Thailand (4.28 tons/ha), Brazil (4.85 tons/ha), and China (5.76 tons/ha). This low agricultural productivity needs to be improved so Indonesia can be more competitive in the global market.

The Indonesian government has made efforts to boost productivity in the agricultural sector through a subsidized credit program for the general public called *Kredit Usaha Rakyat* (KUR) [9]. This is the right step, as a similar program has proven empirically effective. Studies in developing countries such as India [10], Ghana [11], Pakistan [12], and Senegal [13] show identical results, stating that the ability to obtain credit boosts agricultural productivity and technical efficiency. It can help farming households increase their productivity as they can procure more sophisticated technology [14,15] and improve production efficiency as they can procure superior inputs and seeds [16]. The credit also allows farmers to invest in long-term productivity improvement, for example, better irrigation, land preparation, and crop protection [12].

The KUR's realizable value for the agricultural sector continues to increase every year. For example, from 24% in 2016 to 28% in 2020 [17]. However, the program's reach is disproportionately low, which means only a few farmers can enjoy it. Instead, a significant proportion of KUR is absorbed by the trade and retail sector [17]. The World Bank [18] found that only 1.294 million out of 33 million farmers in Indonesia received KUR, indicating difficulty in obtaining loans from financial institutions. This limited access compels farmers to obtain loans from informal sources, such as friends, relatives, input providers, and collectors [19]. Recently, around 30% of Indonesian farmers in rural areas have taken loans from informal sources [20].

Informal credit access indeed has several advantages over formal credit access. For instance, it has low or zero interest rates, customized loan terms and conditions, fewer restrictions on the loan's utilization, and no need for collateral [21,22]. Nevertheless, it also has drawbacks for borrowers, such as a lack of legal protections, prone to exploitative credit practices, and limited borrowing capacity [[23], [24], [25], [26]].

In Indonesia, there have been limited empirical works examining the relationship between the ability to obtain loans and agricultural performances that take into account the source of the credit. Among the few, Ref. [27] employing Tobit regression found that access to credit from banks can increase output and efficiency in cocoa plantations. Ref. [28], utilizing bootstrap truncated regression, also found that access to conventional loans from commercial banks and in-kind credit from farmer groups drive efficiency in several crops' farming, i.e., mangosteen, chili, and shallot. By contrast, loans from microfinance institutions and traders can positively or negatively affect the same crops’ farming. Meanwhile, in oil palm and cocoa plantations, Ref. [29] and Ref. [30], using maximum likelihood estimation and random effect regression, respectively, show that credit access does not significantly influence technical efficiency. From these studies, we can infer that research results on how credit access influences agricultural productivity and efficiency in Indonesia have not been conclusive. It is worth noting that the previous studies applied regression analysis that overlooked the systematic differences between credit and non-credit recipients. Thus, it is more likely that the analysis produced biased estimates.

Against this backdrop, this study evaluates how credit access affects the technical efficiency and productivity of maize farming in Indonesia by considering the sources of credit, i.e., formal and informal. The technical efficiency reflects the best input combinations that produce maximum output and is measured using stochastic frontier analysis (SFA). Meanwhile, productivity is the ratio of total output (kilograms) to land (meter squared). Unlike previous studies, this study applies a quasi-impact evaluation tool, i.e., propensity score matching (PSM), to reduce bias and produce reliable estimates. This method can compare factual and counterfactual groups to estimate a program's impact [31]. Hence, this study enriches the bulk of the literature on the impact of credit access on development in the agriculture sector.

The remainder of this paper is organized as follows. The second section reviews the literature. The third section elaborates on the data and method employed in the analysis. The fourth section is the finding and discussion. The last section is the conclusion.

## 2 Literature review

### 2.1 Productivity and agriculture sector efficiency

The agricultural sector is the driving force of economic development in many countries. Many empirical studies have examined the sector's productivity and efficiency [[32], [33], [34]]. For example, Ref. [33] examined the sector's performance in nine countries in East Asia, showing significant gaps in their productivity growth. The results show decreased total factor productivity (TFP) in all countries due to decreased technical efficiency. Other studies show that socioeconomic factors affect technical efficiency [35,36], which affects TFP. For example, using sample data from 15 provinces in Indonesia, Ref. [36] found that these socioeconomic factors include land size, income, and funding sources.

Access to credit affects agricultural performance. Access to credit can reduce capital constraints and encourage investment in modern technology, which leads to productivity and output growth [37]. Access to credit also increases efficiency [38,39] because it increases the availability of funds needed to meet the input requirements [39], which ultimately increases production and smallholder farmers’ welfare [40]. However, it should be noted that credit may also be detrimental to agricultural performance. A previous study revealed that credit has no significant effect and even reduced efficiency because the money is used unproductively [41].

### 2.2 Access to credit from formal and informal sources

Sources of credit in the agricultural sector may come from formal sources, such as commercial banks, and informal sources, such as brokers, local dealers, informal credit associations, families, or friends. Credit access to financial institutions is an effective tool for increasing agricultural efficiency [12], but there are many barriers for agricultural households to access such credit. Banks will not give credit without sufficient collateral or adequate, stable incomes [42]. As a solution, farmers obtain funding from informal sources, which offer lower interest rates and processing costs and require less collateral [43,44].

Empirical studies have revealed that credit eligibility (both formal and informal) is determined by households’ socio-demographic factors [45], including gender, age, education, farming experience, household income, the existence of credit groups, dependency ratio, size of agricultural land, and ownership certificates [12,42,46]. In several studies, male farmers are considered more creditworthy by formal sources because they often control household resources, so they have higher chances of accessing credit [42,47].

Another factor is education and experience, with higher education and experience gained from farmer field schools. The more educated a farmer is, the better their skills to seek and understand information about credit terms and conditions, assess credit risk, and complete loan application forms correctly [48,49]. The last factor is capital ownership in the form of agricultural land. The larger the farmer's land, the more likely they are to acquire credit because they are perceived as more creditworthy [50]. The agricultural land is often used as collateral in case of repayment failure. Therefore, the bigger the land, the better the credit access is [37,51].

## 3 Methodology

### 3.1 Data and study area

This study applies a quantitative approach using a secondary cross-sectional farming household dataset from the 2014 Secondary Food Crops Survey conducted by Statistics Indonesia (Badan Pusat Statistik/BPS). The results provide (1) national data on secondary food crop commodities, i.e., maize, soybeans, peanuts, green beans, cassava, and sweet potatoes, and (2) information about the agricultural households, including demographics, general agricultural activities, up-to-date information about the crops, production, cost structure, and assets (facilities). Table 1 presents the detailed information of the variables.

**Table 1:** Descriptive statistics.

Variables	Non-credit users (n = 32,922)	Formal credit users (n = 454)		Informal credit users (n = 3141)	
	(1) mean	(2) mean	(3) p-value	(8) mean	(9) p-value
Maize production (kg)	1657.31	4555.93	0.000***	3153.05	0.000***
Fertilizers (kg)	179.40	489.09	0.000***	357.01	0.000***
Seeds (kg)	7.55	16.85	0.000***	13.47	0.000***
Labor (man-days)	27.37	55.34	0.000***	45.04	0.000***
Land (meter squared)	4253.19	9221.00	0.000***	8000.38	0.000***
Land type (1: irrigated land & 0: otherwise)	0.33	0.28	0.022**	0.16	0.000***
Pesticide (1: used pesticide & 0: otherwise)	0.04	0.06	0.000***	0.04	0.019**
Age (year)	50.50	47.48	0.000***	46.59	0.000***
Education (year)	5.39	6.34	0.000***	4.82	0.000***
Sex (1: male & 0: otherwise)	0.89	0.94	0.000***	0.90	0.000***
Farmer field school (1: participated & 0: otherwise)	0.05	0.10	0.000***	0.04	0.000***
Farmer group membership status (1: member & 0: otherwise)	0.46	0.66	0.000***	0.51	0.000***
Land ownership status (1: self-owned & 0: otherwise)	0.73	0.58	0.000***	0.55	0.000***
State electricity (1: power from state enterprise & 0: otherwise)	0.95	0.96	0.106	0.88	0.000***
Non-state electricity (1: power from non-state & 0: otherwise)	0.03	0.02	0.024**	0.07	0.000***
No electricity (1: no power & 0: otherwise)	0.02	0.02	0.749	0.05	0.000***
Floor ceramic (1: floor from ceramic & 0: otherwise)	0.30	0.28	0.331	0.12	0.000***
Floor tile (1: floor from tiles & 0: otherwise)	0.10	0.09	0.562	0.04	0.000***
Floor cement (1: floor from cement & 0: otherwise)	0.33	0.35	0.535	0.42	0.000***
Floor wood (1: floor from wood & 0: otherwise)	0.12	0.15	0.056*	0.27	0.000***
Floor bamboo (1: floor from bamboo & 0: otherwise)	0.01	0.00	0.295	0.01	0.000***
Floor ground (1: floor from land & 0: otherwise)	0.14	0.13	0.547	0.14	0.941
North Sumatera (1: North Sumatera & 0: otherwise)	0.07	0.09	0.117	0.08	0.236
South Sumatera (1: South Sumatera & 0: otherwise)	0.02	0.02	0.913	0.05	0.000***
Lampung (1: Lampung & 0: otherwise)	0.05	0.04	0.229	0.07	0.000***
West Java (1: West Java & 0: otherwise)	0.06	0.01	0.000***	0.04	0.000***
Central Java (1: Central Java & 0: otherwise)	0.23	0.14	0.000***	0.08	0.000***
East Java (1: East Java & 0: otherwise)	0.35	0.40	0.02**	0.14	0.000***
West Nusa Tenggara (1: West Nusa Tenggara & 0: otherwise)	0.03	0.16	0.000***	0.11	0.000***
North Sulawesi (1: North Sulawesi & 0: otherwise)	0.05	0.02	0.02**	0.03	0.000***
South Sulawesi (1: South Sulawesi & 0: otherwise)	0.09	0.05	0.000***	0.27	0.000***
Gorontalo (1: Gorontalo & 0: otherwise)	0.03	0.04	0.617	0.13	0.000***

*Note: statistical mean differences between the treatment and control group (unmatched sample) are denoted by the p-value.* ***p < 0.01, **p < 0.05, *p < 0.10.

**Fig. 1:**
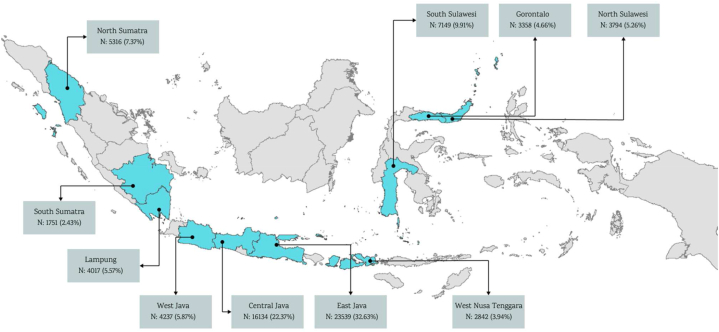
Study area map.

The study area focused on maize farming households located in ten largest maize-producing provinces in Indonesia: (Sumatera Island: North Sumatra, South Sumatra, Lampung); (Java Island: West Java, Central Java, East Java); (Nusa Tenggara Island: West Nusa Tenggara); (Sulawesi Island: North Sulawesi, South Sulawesi, and Gorontalo). The total sample studied was 36,157 maize farming households with the distribution across provinces as follows: North Sumatera (5316 households/7.37%), South Sumatra (1751 households/2.43%); Lampung (4017 households/5.57%); West Java (4237 households/20%); Central Java (16,134 households/22.37%); East Java (23,539 households/32.63%); West Nusa Tenggara (2842 households/3.94%); North Sulawesi (3794 households/5.26%); South Sulawesi (7149 households/9.91%); and Gorontalo (3358 households/4.66%). Fig. 1 illustrates the study location.

### 3.2 Farming households survey by statistics Indonesia (BPS)

Statistics Indonesia (BPS) collected the data through a structured questionnaire administered in the study area. Statistics Indonesia employed two sampling frames called census block and household sampling frame. The sample frame of census block selection is determined by the list of ordinary census blocks and preparation census blocks containing households that are stratified based on the type of secondary food crops cultivated by households during the last year. An eligible census block is a block that has a minimum of ten or more eligible households. Additionally, the sampling frame for household selection is determined based on the secondary crop production updated in every selected census block, and it was ordered by the primary type of secondary crop.

The survey employed a two-stage sampling method. The first stage uses the probability proportional to size method to select census blocks from the census block sampling frame. The second stage uses systematic random sampling to select the eligible farming households from the households sampling frame. A farming household is eligible if it has a minimum harvested area of 1500 m^2^ [7].

### 3.3 Agricultural productivity and efficiency

This study assesses the impact of credit access on performance in the agriculture sector, proxied by productivity and technical efficiency, which indicates the skills and ability to utilize different inputs in a production process to generate a maximum output [52]. Technical efficiency is measured using stochastic frontier analysis (SFA) as the parametric approach and *data envelopment analysis* (DEA) as the non-parametric approach. It is important to note that DEA has a few shortcomings: (1) treating deviations from the production frontier as inefficiencies, (2) assuming no stochastic error, and (3) being sensitive to outliers. As such, DEA is unsuitable for measuring efficiency in agricultural activities as it is influenced by uncontrolled factors, such as unpredictable weather, droughts, and storms [53].

SFA has some advantages over DEA, as pointed out by Ref. [54]: (1) it can measure efficiency and estimate the causes in one stage of analysis, whereas DEA requires two stages; (2) SFA can separate the efficiency of the unit of analysis from the stochastic variation at the frontier. This is possible because SFA assumes deviations from the production frontier not only come from the inefficiency of a component but can also come from the noise term. Thus, variables beyond a producer's control, such as natural disasters and crises, are captured in the technical efficiency measurement [55,56].

According to Ref. [57], in SFA, productivity is a ratio of output to the input used at a certain production level (a production frontier). Meanwhile, technical efficiency is the ratio of the maximum possible output and the input used at a certain point of production technology. The mathematical function to represent SFA is as follows.

(1) $$Y_{i}=f\left(X_{i},\beta \right)+\varepsilon_{i};\;\varepsilon_{i}=v_{i}-u_{i}$$

where $Y_{i}$ represents the output variable, $X_{i}$ is the input notation, and β is the estimated parameter. Meanwhile, $\varepsilon_{i}$ is an error term with two elements: $v_{i}$ (random error) and $u_{i}$ (inefficiency error). $v_{i}$ is expected to be independent and identically distributed as $N\left(0,\sigma^{2}\right)$. Meanwhile, $u_{i}$ has a non-negative value with an asymmetric distribution [56].

Equation (1) requires the functional specification of f(.) to show the Douglas function specification, Ref. [53,58,59] denoted as follows:

(2) $$\mathrm{Ln}\left(Y_{i}\right)=\beta_{0}+\sum_{j=1}^{6}\beta_{j}\;\mathrm{Ln}\;X_{i}+v_{i}-u_{i}$$

where $Y_{i}$ is corn production (kg) from the $i^{th}$ farmer and $X_{i}$ represents the input used. The inputs used in the stochastic model are fertilizer, seeds, workers, and land. Dummy variables are also included for the use of pesticides and land type based on the type of irrigation. $v_{i}$ and $u_{i}$ are composite errors. All input and output variables are transformed into natural logarithmic form, except for dummy variables $\beta_{0}$ dan $\beta_{j}$, which are a constant and a parameter with values yielded from the estimation results. Table 1 shows the input and output in more detail.

After obtaining the production frontier based on equation (2), each $i^{th}$ farmer's technical efficiency is measured by the ratio of actual and estimated output of the frontier to the amount of input as indicated in equation (3). Specifically, the technical efficiency function can be denoted as follows.

(3) $${TE}_{i}=\frac{Y_{i}}{Y_{i}^{*}}$$

where $Y_{i}$ denotes the real output, and $Y_{i}^{*}$ is the frontier's estimated output. Efficiency is reached (TE=1) if the actual output is on the production frontier. If the actual output falls below the frontier's estimated output, then farmer is classified as inefficient.

### 3.4 Access to formal and informal Credit's effect on productivity and technical efficiency

The decision to acquire formal or informal credit is an individual choice. The decision to acquire from a formal institution over an informal one, and vice versa, may also affect technical efficiency and productivity. This selection bias (self-selection) comes from observed and unobserved factors [53], which can be corrected by propensity score matching (PSM) so that the analysis results remain robust. Ref. [56] explained that PSM could effectively estimate credit impact by comparing the treatment group's observable outcomes and the counterfactual control group's outcome. PSM can also be used to evaluate the impact of a program without using longitudinal data, as was done in this study.

Moreover, PSM in this study also ensures that the farmers in the treatment group (those accessing formal and informal credit) and those in the control group (those not accessing credit) match. Then, the average difference in the outcome of the two groups is estimated. PSM can create similar groups as the counterfactuals by estimating the pre-treatment variables' propensity score, which indicates the probability of participation). The function of the propensity score is denoted in equation (4).

(4) $$p\left(X\right)=\mathrm{Pr}\left[T=1|X\right];p\left(X\right)=F\left\{h\left(X_{i}\right)\right\}$$

where p(X) is the propensity score, and Pr is the possibility of accessing formal and informal credit. (T=1) represents farmers who access formal and informal credit, and (T=0) represents farmers with no access credit at a certain X, a vector of the observed covariance. F{.} suggests the probability distribution which is logistic distribution (logit).

After obtaining the propensity scores of farmers with the ability to obtain formal or informal credit, we use a Kernel and Radius Caliper matching algorithm to compare them with those without credit access. The fulfillment of several conditions ensured the reliability of the matching results: (1) the matching algorithm does not remove observations excessively from the final stage of the analysis, and (2) there is a balance of average covariates when the treatment group is compared to the control group; and the fulfillment of the conditional independence assumptions [60,61]. Therefore, we ran a statistical *t*-test to assess the balance of the covariates between the groups. We also employ the Rosenbaum sensitivity analysis test to check the assumption of conditional independence assumption, making sure potential confounders cause no hidden bias.

Then, the causal impact of access to formal and informal credit on farmer outcomes (productivity and technical efficiency) is calculated using equation (5).

(5) $${ATT}^{PSM}=E\left[Y\left(1\right)|T=1,p\left(X\right)\right]-E\left[Y\left(0\right)|T=0,p\left(X\right)\right]$$

where T indicates information on access to formal and informal credit. The values are T = 1 if the farmer can secure the credit and T = 0 otherwise. Y(1) is an outcome indicator for farmers with credit access, and Y(0) is an outcome indicator for farmers without credit access. Meanwhile, p(X) is the propensity score.

## 4 Findings and discussion

### 4.1 Descriptive statistics

Table 1 shows the variables' descriptive statistics in the analysis. The data is categorized based on the types of credit that farmers can access. As many as 3595 farmers had access to credit (treatment group), with 12.62% acquiring it from formal sources and 87.37% from informal sources. Farmers with no credit access are the control group in the analysis. In general, the treatment group's maize production is higher. Farmers with formal credit access produced 4555.93 kg, and farmers with informal credit access produced 3153.05 kg. Meanwhile, the average production of the control group is 1657.31 kg. Farmers with the ability to obtain credit also use more fertilizer (an average of 489.09 kg and 357.01 kg by those accessing formal and informal credit, respectively). Meanwhile, farmers who cannot access credit use only 179.40 on average. Likewise, the average use of seeds is higher among farmers with access to credit (16.85 kg and 13.47 kg by those accessing formal and informal credit, respectively). The land use is also substantially bigger among farmers with access to credit from formal and informal sources, an average of 9221.0 and 8000.38 square meters, respectively). Meanwhile, farmers with no credit access cultivate nearly half as wide, i.e., 4253.19 square meters. However, the average land use for irrigation by farmers with credit access is lower (28% and 16%) than those without access (33%). Lastly, the use of pesticides by farmers with access to formal credit is higher (6%) than by farmers with no access or with access to informal credit (4%).

As for the demographics, farmers who can access credit are younger (47.48 years and 46.59 years among those accessing credit from formal and informal sources, respectively). Meanwhile, farmers who cannot access credit are aged 50.50 years on average. As for education, farmers who can access formal credit are more educated (6.34 years) than those with no access (5.39 years) or those with access to informal credit (4.82 years). Most of the farmers in both groups are men. Only a few attend farmer field schools: 10% of those with access to credit from formal sources, 4% of those with credit from informal sources, and 5% of those with no access. Most are members of farmers’ groups: 66% of those with access to formal credit, 51% with access to informal credit, and 46% without access. Regarding land ownership, 58% and 55% of farmers who access credit from formal and informal sources, respectively, have land in their names. Meanwhile, 73% of those without access have land ownership registered in their names.

### 4.2 Technical efficiency estimation

The production model using stochastic frontier measures technical efficiency by estimating the production function [62]. Table 2 presents the two models' maximum likelihood estimates of the Cobb-Douglas stochastic production function. Models (1) and (2) estimate input variables in maize production by farmers with access to formal and informal credit, respectively. Each model includes a dummy province.

**Table 2:** Parameter calculation of stochastic production function.

	(1)	(2)
VARIABLES	Formal	Informal
Fertilizer	0.2083***	0.2087***
	(0.0042)	(0.0040)
Seeds	0.1028***	0.1098***
	(0.0060)	(0.0058)
Labor	0.1441***	0.1435***
	(0.0055)	(0.0052)
Land	0.5909***	0.5828***
	(0.0077)	(0.0073)
Dummy pesticide	0.0439***	0.0358***
	(0.0133)	(0.0127)
Dummy land type	0.1555***	0.1525***
	(0.0060)	(0.0058)
Constant	1.0550***	1.1079***
	(0.0420)	(0.0404)
Dummy Province	YES	YES
Diagnostic Statistics		
Sigma_*v*	0.3223*** (0.0039)	0.3205*** (0.0037)
Lambda	1.5022*** (0.0089)	1.5042*** (0.0084)
Wald chi2	102,653.41	114,174.38
Prob > Chi2	0.0000	0.0000
Log Likelihood Function	−26,560	−28,520
Observations	33,376	36,063

Standard errors in parentheses.

***p < 0.01, **p < 0.05, *p < 0.1.

The estimation results show that fertilizers, seeds, labor, and land (input variables) positively affect maize production. The coefficients of all variables show a significant effect on maize production in both groups of farmers, which is in line with research by Refs. [63,64]. The nature of the estimated production input coefficient is partial elasticity. For example, an increase in 1% fertilizer input will boost maize production by 0.2083% among farmers with access to formal credit and by 0.2087% among those with access to informal credit, ceteris paribus. These results align with studies by Refs. [39,62].

Meanwhile, the effect of the pesticide dummy variable is significant, with a coefficient of 0.0439 and 0.0358 for farmers with access to formal and informal credit, respectively. Using pesticides, on average, improves production by 4.39% and 3.58% than not using pesticides. This finding aligns with research by Ref. [65], stating that farmers can increase their output and productivity by applying fertilizers and pesticides because soil nutrients will be filled with nitrogen and phosphorus. The same applies to the dummy variable land type (irrigation). On average, cultivation on irrigated land produces 15.55% more maize among farmers with access to formal credit and 15.25% for those with access to informal credit. These results align with research by Ref. [66], stating that irrigation is crucial in rice cultivation as it allows plants to develop properly.

### 4.3 Determinant of credit access

Factors influencing farmers' credit access decisions are calculated using logistic regression. Table 3 presents the results, showing that various factors impact the decision to access formal and informal credit. Variables significantly determining farmers’ credit access from formal sources are age, gender, farmer field school attendance, farmer group membership, farm size, farm size squared, land ownership, non-state electricity, and housing floor type (wood). Meanwhile, credit access from informal sources is associated with age, education, farmer field school attendance, farmer group membership, farm size, farm size squared, land ownership, state electricity, and housing floor types (cement, wood, bamboo, and ground).

**Table 3:** Logistic regression of credit access.

	(1)	(2)
VARIABLES	Formal	Informal
Age	−0.0147***	−0.0162***
	(0.0046)	(0.0018)
Education	0.0168	−0.0509***
	(0.0128)	(0.0054)
Gender	0.3673*	−0.0311
	(0.2070)	(0.0697)
Farmer field school	0.3582**	−0.3650***
	(0.1667)	(0.1041)
Farmer group membership	0.6605***	0.2329***
	(0.1053)	(0.0414)
Farm size	0.0002***	0.0001***
	(0.0000)	(0.0000)
Farm size squared	−0.0000***	−0.0000***
	(0.0000)	(0.0000)
Land ownership	−0.6307***	−0.7730***
	(0.1006)	(0.0417)
State electricity	0.0755	0.2129**
	(0.3402)	(0.0994)
Non-state electricity	−1.1127**	0.0140
	(0.5081)	(0.1227)
Floor made of tiles	0.2121	0.0426
	(0.1865)	(0.1131)
Floor made of cement	0.1975	0.5856***
	(0.1334)	(0.0676)
Floor made of wood	0.6287***	0.5619***
	(0.1922)	(0.0804)
Floor made of bamboo	−0.4189	0.7672***
	(1.0224)	(0.1994)
Floor made of ground	0.0971	1.0447***
	(0.1649)	(0.0791)
Constant	−4.7200***	−2.8609***
	(0.4856)	(0.1722)
		
Dummy provinces	YES	YES
		
Observations	33,376	36,063
Pseudo R-Squared	0.1280	0.1693

Standard errors in parentheses ***p < 0.01, **p < 0.05, *p < 0.1.

The estimated age coefficient negatively and significantly impacts the decision to obtain either formal or informal credit. The older the farmer, the lower the chances of accessing credit. This finding aligns with previous research by Ref. [56], where younger farmers tend to be more receptive to new technologies and innovative activities that would generate income for them. In addition, most financial sources may not approve older farmers' credit proposals, fearing they may be unable to complete the payment. Older farmers may also be considered high-risk clients with low productivity by financial institutions [40]. This finding contrasts the work of Ref. [67], which stated that older farmers’ probability of securing credit is higher.

Farmer education does not affect the decision to access formal credit but negatively and significantly affects access to informal credit. The negative estimation coefficient means the higher the farmer's education, the lower the chances of accessing credit from informal sources. Research by Ref. [43] found that farmers with secondary education could obtain information about funding sources and farming technologies better. In addition, higher education levels imply a better understanding of using superior techniques to increase productivity so that they may repay credit immediately. In other words, more educated farmers are less associated with agricultural credit. They are likely to be more productive, allowing them to raise capital for farming.

As for agricultural field school attendance, the estimates show a positive and significant impact on access to formal credit. This finding confirms the research by Ref. [44], stating that farmer education is significantly and positively linked to farmer access to credit. More educated farmers access credit from formal sources than informal sources [68].

Gender is another important variable considered in the analysis. The estimation results show that gender only affects access to informal credit significantly, with male farmers having better access. This finding aligns with research by Refs. [42,47], stating that male farmers are more creditworthy by formal lenders because they are in charge of household resources, so they tend to secure credit more easily than women.

Farmer group participation also determines the decision to obtain credit. The estimation results show that participation positively and significantly affects access, meaning that farmer group members have higher access to formal or informal credit. This finding aligns with research by Ref. [42], stating that households with more social interaction and networking, such as a farmer group, are more likely to access credit. Farmer groups can also help secure, distribute, and repay funds, as well as obtain lower interests and make loans safer [69].

The size of agricultural land positively and significantly affects credit access, both from formal and informal sources. This suggests that an increase in farmers’ land increases credit access, which confirms the results in past research, stating that the larger the land, the more input is needed, hence the higher the loans [12]. By contrast, smaller land increases transaction costs, disincentivizing lenders and burdening farmers with higher credit costs [43], resulting in lower access. Larger land makes farmers more creditworthy [50] because they can take advantage of the economies of scale and repay the credit [49]. Meanwhile, the squared land area coefficient is negative, indicating a parabolic relationship between land size and credit access.

Land ownership negatively and significantly affects both formal and informal credit access, indicating that farmers who own land tend not to access credit. In terms of electricity sources, state electricity positively and significantly affects access to informal credit, and non-state electricity negatively and significantly affects formal credit access. This shows that the possibility of farmers accessing formal credit is lower if the electricity comes from the state. In other words, they tend to access credit from informal sources.

Meanwhile, house floor types have different effects. The tiled floor has no effect, but the cement floor positively and significantly affects access to informal credit. This means that farmers with housing built from cement show a greater possibility of accessing informal credit. As for the wood floor variable, it positively and significantly affects credit from both sources. Lastly, the bamboo floor and ground floor variables positively and significantly affect access to informal credit.

### 4.4 The impact of credit access on productivity and technical efficiency

The PSM is estimated using a logit regression model, where the treatment indicator (farmers accessing formal and informal credit) is influenced by several socioeconomic characteristics listed in Table 3. Then, the results of logistic regression, i.e., the predicted probability of accessing formal and informal credit, are used to predict the impact of credit access from both sources on the productivity and technical efficiency of maize production. In this case, the treatment group's average productivity and technical efficiency are compared with those of the control group with identical propensity scores.

**Fig. 2:**
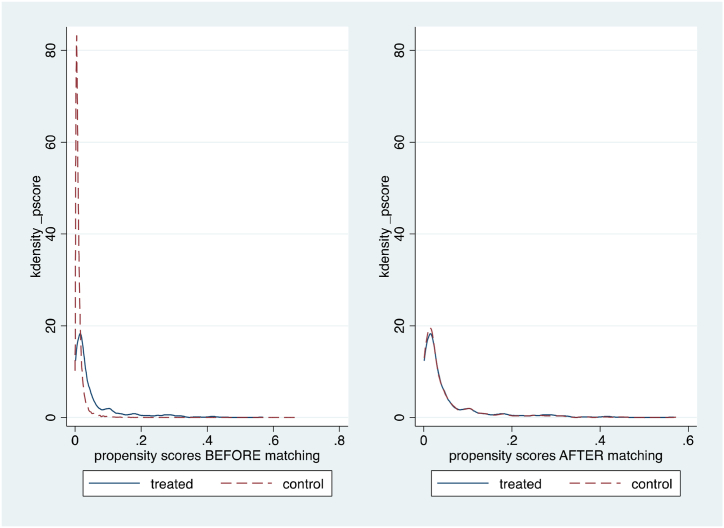
Distribution of estimated propensity scores across treatment (credit access from formal sources) and control groups before and after matching.

Fig. 2 illustrates the propensity score distribution between farmers who can access formal credit (treatment group) and those who cannot (control group) pre- and post-matching. The overlap in the distribution after matching indicates a substantial range of common support. Table 4 shows the covariate balance test between farmers who can access formal credit (treatment group) and those who cannot (control group). The matching between the treatment and control groups shows balanced observed characteristics. Thus, it can be concluded that the difference is not statistically significant between farmers who can access formal credit and farmers without credit access after matching.

**Table 4:** After matching quality test for formal credit.

Variables	Mean		*p*-value
	Group of Formal Credit receipt	Group of non-credit receipt	
**Age**	47.48	48.11	0.400
**Education**	6.33	6.42	0.738
**Gender**	0.94	0.93	0.779
**Farm field school**	0.10	0.10	1.000
**Farmer group membership**	0.65	0.62	0.333
**Land ownership**	0.58	0.57	0.788
**State electricity**	0.96	0.96	1.000
**Non-state electricity**	0.01	0.01	1.000
**No electricity**	0.02	0.02	1.000
**Floor made of ceramic**	0.28	0.29	0.769
**Floor made of tiles**	0.08	0.10	0.370
**Floor made of cement**	0.34	0.36	0.678
**Floor made of wood**	0.14	0.13	0.703
**Floor made of bamboo**	0.00	0.00	1.000
**Floor made of ground**	0.13	0.10	0.152

Note: statistical mean differences between the group (matched sample) are indicated by the p-value. ***p < 0.01, **p < 0.05, *p < 0.1.

**Fig. 3:**
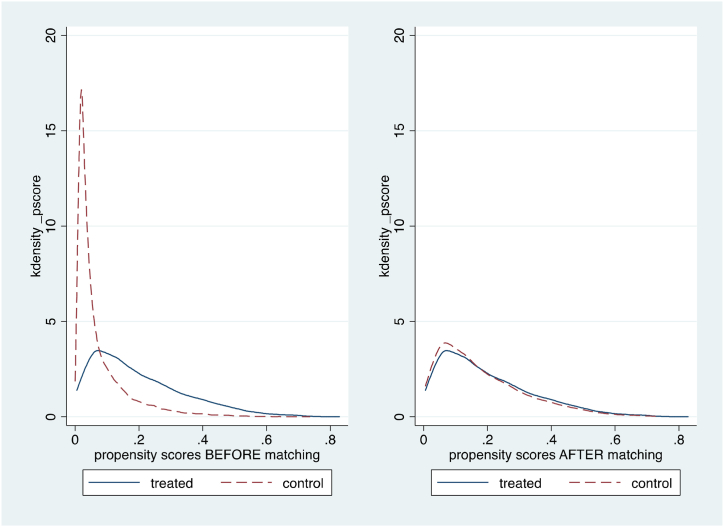
Distribution of estimated propensity scores across treatment (credit access from informal sources) and control groups before and after matching.

Fig. 3 illustrates the propensity score distribution between farmers who access informal credit (treatment group) and farmers with no credit access (control group) before and after matching. Table 5 shows the covariate balance test between farmers who access credit from informal sources (treatment group) and farmers with no credit access (control group). It can be concluded that the difference between the treatment and control group is not statistically significant after matching.

**Table 5:** After matching quality test for informal credit.

Variables	Mean		*p*-value
	Group of Informal Credit receipt	Group of non-credit receipt	
Age	46.59	46.92	0.267
Education	4.82	4.84	0.771
Gender	0.90	0.92	0.148
Farm field school	0.03	0.03	0.311
Farmer group membership	0.51	0.50	0.705
Land ownership	0.55	0.55	0.980
State electricity	0.87	0.88	0.368
Non-state electricity	0.06	0.06	0.389
No electricity	0.05	0.05	0.731
Floor made of ceramic	0.11	0.12	0.585
Floor made of tiles	0.03	0.03	0.369
Floor made of cement	0.42	0.42	0.683
Floor made of wood	0.26	0.25	0.510
Floor made of bamboo	0.01	0.01	0.653
Floor made of ground	0.14	0.14	0.943

Note: statistical mean differences between the group (matched sample) are indicated by the p-value. ***p < 0.01, **p < 0.05, *p < 0.1.

In addition to the substantial range of common support, it is essential to check the conditional independence assumption by conducting the Rosenbaum sensitivity analysis test. Table 6 reports the result of the Rosenbaum sensitivity analysis test.

**Table 6:** Rosenbaum sensitivity analysis test.

Matching Algorithm	Sensitivity (*Γ*)	
	Efficiency	Productivity
**Formal Source Credit**		
Kernel Matching Bandwidth	2.5	2.2
Radius Caliper	2.4	2.1
**Informal Source Credit**		
Kernel Matching Bandwidth	1.5	2.2
Radius Caliper	1.5	2.1

The critical value of *Г* with a significance level of α = 0.05 for formal credit access ranges from 2.1 to 2.5. It means that if the odds of a farmer receiving formal credit access are 2.1–2.5 times higher due to differences in unobserved covariate values, regardless of being identical on matched covariates, the inference will change. It indicates that our finding on the impact of formal credit access is not sensitive to the issue of hidden bias. The critical value of *Г* for informal credit access was around 1.5 to 2.2. It shows that if the odds of the farmer obtaining informal credit are 1.5–2.2 times higher, caused by differences in unobserved covariate values despite being similar to the matched covariates, the inference will change. Therefore, our finding on the impact of informal credit access seems insensitive to the potential of hidden bias by unobserved variables. Some studies reported that the critical value of *Г*, which ranges from 1.3 to 1.7, is relatively sufficient to indicate the absence of hidden bias caused by unobservable variables [[70], [71], [72]].

Table 7 presents the PSM estimates of the impact of access to credit on agriculture performance by using two matching algorithms: Kernel and Radius Caliper algorithm.^1^ In general, credit access from any source positively and significantly impacts technical efficiency and productivity. The matched sample shows that farmers with formal credit access show a higher technical efficiency score of 0.0571 than those with credit access. Meanwhile, farmers with informal credit access have a higher technical efficiency score of 0.0163 than farmers without credit access. This study's findings align with previous research by Refs. [12,73]. Ref. [12] analyzed the influence of credit access on the technical efficiency of maize farming in Ghana. The results show a positive correlation between technical efficiency and agricultural credit. This is because access to financial services is vital in increasing efficiency and productivity in agriculture due to the ability to make long-term investments, as shown by Ref. [73]. In addition, credit availability increases farmer liquidity, thereby increasing access to new technologies and inputs [56,74]. Therefore, Ref. [56] states that efficiency gains can be maintained through stronger partnerships with financial sources.

**Table 7:** Impact of formal credit and informal credit on efficiency and productivity.

Variables	Kernel Algorithm		Radius caliper Algorithm	
	Formal	Informal	Formal	Informal
Efficiency	0.0571*** (0.0075)	0.0163*** (0.0037)	0.0529*** (0.0076)	0.0166*** (0.0037)
Productivity	0.0929*** (0.0101)	0.0278*** (0.0041)	0.0880*** (0.0102)	0.0281*** (0.0042)

Standard Errors in parentheses ***p < 0.01, **p < 0.05, *p < 0.1.

Productivity is another indicator to measure the performance in agriculture. The results of the propensity score estimation show that credit access from any source positively affects maize agricultural productivity. The matched sample shows that farmers with formal credit access have a higher productivity of 0.0929 than farmers without credit access. Meanwhile, farmers with formal credit access have a higher productivity of 0.0278 than farmers without credit access. This finding confirms past research by Refs. [50,75], stating that access to credit is fundamental in increasing agricultural productivity. With access to credit, households can procure more productive and modern hybrid varieties, which will bring about changes in the production frontier [16].

Overall, the impact of formal credit access on agricultural performance is more significant than the informal one. This finding aligns with a previous study by Refs. [76,77]. However, these findings contradict Ref. [78] research, showing that formal credit's effect on agricultural productivity is lower than informal credit, arguably because informal lenders provide credit in accordance with the borrower's socioeconomic and cultural circumstances.

However, studies have also shown that agricultural credit may not be effectively used [79]. Instead, it is used to purchase consumer goods and services. Furthermore, credit from informal sources is mainly used to meet farmers’ basic needs (e.g., healthcare, education, housing, and social activities), whereas credit from formal financial sources is often invested in crop production [76]. Based on the Survey on Financial Inclusion and Access, the amount of loans provided by formal financial sources is greater than that of informal sources [80]. Therefore, the impact of credit from informal loans tends to be negligible on agricultural productivity and efficiency.

## 5 Conclusion

This study examines the causal impact of access to formal and informal credit on agricultural performance proxied by productivity and efficiency. Unlike previous studies, this study uses a more robust method, PSM, which can correct selection bias in the impact of credit access. This study contributes to the literature by evaluating the impact of access to credit on productivity and efficiency in Indonesia by taking into account the source of the credit (formal and informal credit access), which has never been done before.

Based on the impact evaluation results, formal and informal credit positively and significantly impacts efficiency and productivity. However, this study found that access to formal credit impacts efficiency and productivity scores more than access to informal credit. In other words, formal credit is more urgent than informal credit in increasing the efficiency and productivity of maize agriculture in Indonesia.

The implication is that providing credit to farmers must be a vital component of a strategy to increase agricultural productivity and efficiency in Indonesia. Given the strong influence of formal credit, fostering partnerships with financial sources is needed to boost productivity and efficiency. Policy interventions to increase farmers’ access to formal credit should be prioritized, which can be done by creating a conducive investment environment and lowering loan interest rates and collateral requirements. Finally, this current research has some drawbacks: the employment of a relatively outdated dataset and the focus on one agricultural commodity, i.e., maize only. Future research might employ a recent dataset and explore the impact of credit access on other agricultural commodities. Furthermore, it is also essential to examine the impact of credit access on agricultural performance using experiment settings: laboratory or field experiment.

## Author contribution statement

Tri Haryanto: Conceived and designed the analysis; Wrote the paper. Wahyu Wisnu Wardana: Analyzed and interpreted the data; Wrote the paper. Iqram Ramadhan Jamil: Analyzed and interpreted the data; Contributed analysis tools or data. Annisaa Rizky Dwi Brintanti: Analyzed and interpreted the data; Contributed analysis tools or data. Kabiru Hannafi Ibrahim: Contributed analysis tools or data; Wrote the paper.

## Funding Statement

This work was supported by the Direktorat Riset, Teknologi, dan Pengabdian Masyarakat Kementerian Pendidikan, Kebudayaan, Riset dan Teknologi Republik Indonesia.

## Declaration of competing interest

The authors declare that they have no known competing financial interests or personal relationships that could have appeared to influence the work reported in this paper.

## Footnotes

1 The result of Kernel algorithm is used as the basis of analysis.
